# Characterization of the Potential Role of NTPCR in Epithelial Ovarian Cancer by Integrating Transcriptomic and Metabolomic Analysis

**DOI:** 10.3389/fgene.2021.695245

**Published:** 2021-09-01

**Authors:** Hongkai Shang, Huizhi Zhang, Ziyao Ren, Hongjiang Zhao, Zhifen Zhang, Jinyi Tong

**Affiliations:** ^1^Department of the Fourth Clinical Medical College, Zhejiang Chinese Medical University, Hangzhou, China; ^2^Department of Gynecology, Hangzhou First People’s Hospital, Hangzhou, China; ^3^Department of Gynecology, Zhejiang University School of Medicine, Hangzhou, China; ^4^Department of Gynecology, Hangzhou Women’s Hospital (Maternity and Child Health Care Hospital), Hangzhou, China

**Keywords:** epithelial ovarian cancer, transcriptome sequencing, metabolomics sequencing, NTPCR, nucleoside-triphosphatase cancer-related

## Abstract

**Background:**

Epithelial ovarian carcinoma (EOC) is a malignant tumor with high motility in women. Our previous study found that dysregulated nucleoside-triphosphatase cancer-related (NTPCR) was associated with the prognosis of EOC patients, and thus, this present study attempted to explore the potential roles of NTPCR in disease progression.

**Methods:**

Expressed level of NTPCR was investigated in EOC tissues by RT-qPCR and Western blot analysis. NTPCR shRNA and overexpression vector were generated and transfected into OVCAR-3 or SKOV3 cells to detect the effect of NTPCR on cell proliferation, cell cycle, cell migration, and invasion. Transcriptomic sequencing and metabolite profiling analysis were performed in shNTPCR groups to identify transcriptome or metabolite alteration that might contribute to EOC. Finally, we searched the overlapped signaling pathways correlated with differential metabolites and differentially expressed genes (DEGs) by integrating analysis.

**Results:**

Comparing para-cancerous tissues, we found that NTPCR is highly expressed in cancer tissues (*p* < 0.05). Overexpression of NTPCR inhibited cell proliferation, migration, and invasion and reduced the proportion of S- and G2/M-phase cells, while downregulation of NTPCR showed the opposite results. RNA sequencing analysis demonstrated cohorts of DEGs were identified in shNTPCR samples. Protein–protein interaction networks were constructed for DEGs. STAT1 (degree = 43) and OAS2 (degree = 36) were identified as hub genes in the network. Several miRNAs together with target genes were predicted to be crucial genes related to disease progression, including *hsa-miR-124-3p*, *hsa-miR-30a-5p*, *hsa-miR-146a-5*, *EP300*, *GATA2*, and *STAT3*. We also screened the differential metabolites from shNTPCR samples, including 22 upregulated and 22 downregulated metabolites. By integrating transcriptomics and metabolomics analysis, eight overlapped pathways were correlated with these DEGs and differential metabolites, such as primary bile acid biosynthesis, protein digestion, and absorption, pentose, and glucuronate interconversions.

**Conclusion:**

NTPCR might serve as a tumor suppressor in EOC progression. Our results demonstrated that DEGs and differential metabolites were mainly related to several signaling pathways, which might be a crucial role in the progression of NTPCR regulation of EOC.

## Introduction

Epithelial ovarian carcinoma (EOC) is a leading cause of cancer death and accounts for a high incidence rate in women more than 50 years old. Surgery and platinum-based chemotherapy have been confirmed as a standard treatment method for advanced EOCs. However, the 5 years survival rate of advanced EOC is still less than 30% for platinum resistance or disease relapse (Holschneider and Berek, 2000). Thus, understanding the mechanism of disease progression will promote an effective therapeutic method for EOC.

As the cost becomes more affordable in next-generation sequencing (NGS) technology, transcriptomic analysis has been extensively utilized to identify novel biomarkers for early detection, diagnosis, and therapeutics in cancers. A recent study of systematic transcriptome analysis has revealed tumor-specific mRNA isoforms for ovarian cancer diagnosis and therapy by using RNA-seq methods (Barrett et al., 2015). Another paper showed NGS was used to identify transcriptome changes and hypermethylation associated with cisplatin resistance in high-grade serous ovarian cancer (Lund et al., 2017). Although findings promoted a better understanding of ovarian cancer, disease progression still could not be explained only on the transcriptomic alteration. More efforts should be directed to elucidate complicated genetic network and its interactions with biomolecules, such as metabolites. Recently, integrating transcriptome profiling with metabolic profiling analysis has been demonstrated to be an effective method to identify differential genetic and metabolic pathways in various cancer types (Zhang G. et al., 2013; Popławski et al., 2017).

Our previous study has reported that high expression levels of nucleoside-triphosphatase cancer-related (NTPCR) were related to better prognosis in EOC patients (Shang et al., 2020). Thus, this present study aimed to explore the potential roles of NTPCR in disease progression. Based on transcriptomic profiling analysis, we screened differentially expressed genes (DEGs) between shNTPCR groups and shNC groups in EOC cells, and following performed functional analysis, interaction network analysis, prediction of miRNAs, and target transcription factors (TFs). Integrating the transcriptomic and metabolomic profiling analysis revealed crucial overlapped pathways correlated with DEGs and metabolites in EOC. The integrative analysis of our study promoted a better understanding of NTPCR roles in EOC, which might facilitate therapeutic or prognosis discovery of biomarkers.

## Materials and Methods

### Cell Lines and Culture Conditions

Epithelial ovarian carcinoma cell lines (SKOV3, CAOV-3, OV-1063, and OVCAR-3) and human ovarian epithelium cell line IOSE80 were purchased from Chinese Academy of Sciences (Beijing, China). The cells were cultured in RPMI-1640 medium supplement with 10% fetal bovine serum and maintained in a condition of 37°C and 5% CO_2_.

### Samples

Approved by the Ethics Committee of Hangzhou First People’s Hospital (approval no. IRB#2021-20210406-01), with informed consent by patients, we collected 22 tissue samples from 11 patients. These specimens were human tissue specimens that were pathologically diagnosed as epithelial ovarian cancer during staging ovarian cancer surgery, including cancerous tissues and para-cancerous tissues.

### RT-PCR Analysis

The total RNA was extracted from EOC cells and tissues using TRIzol (TaKaRa Inc. Tokyo, Japan). Reverse transcription kit (TaKaRa Inc., Tokyo, Japan) was used to transform RNA to cDNA. The mRNA levels of NTPCR in tumor cells and tissues were detected using SYBR Green kit (Thermo Inc., Waltham, MA, United States), and GAPDH was set as an endogenous control. The relative expression levels of NTPCR in different samples were determined by the method of comparing CT values (ΔΔCT) and normalization with internal reference. The primers were NTPCR-hF: ACCCGTCTTGAGGAATGTGA and NTPCR-hR: CTCTTGAACTGGGCACTCCT, and GAPDH-hF: TGACAACTTTGGTATCGTGGAAGG and GAPDH-hR: AGGCAGGGATGATGTTCTGGAGAG.

### Cell Transfection

The overexpression vector of NTPCR used a commercial vector (Sino Biological, Beijing, China, cat: HG17161-NY); shRNA was synthesized by Shanghai Gima Biopharmaceutical Technology (sequence as follows): shRNA1, 5′-GGCCTTTATCGAGAGTTGGGTTAGA-3′; shRNA2, 5′-CCTCTGGTGTGCCTGTTGATGGATT-3′; and shRNA3, 5′-CAGTATGTGGTCGACCTGACTTCTT-3′. shRNA was inserted into pGPU6/Neo to obtain the corresponding plasmid. SKOV3 and OVCAR-3 cells were taken in the logarithmic growth phase and transfected with Lipofectamine^TM^ 2000. Six hours later, the mixture was aspirated and changed to a normal medium to continue culturing. After 72 h, the cells were harvested and used to detect molecular expression by RT-PCR.

### Cell Viability

Cell viability was evaluated using a CCK-8 kit (Beyotime Biotechnology, China) following the protocol of the manufacturers. SKOV3 and OVCAR-3 cells were seeded into 96-well plates with 3,000 cells per well. After transfecting the overexpression vector or interference shRNA vector of NTPCR for 24, 48, 72, and 96 h, a total of 10 μl CCK-8 solution was added to wells, and the optical density (OD) values were read at 450 nm using a microplate reader system.

### 5-Ethynyl-2′-deoxyuridine (EdU)

Cell proliferation was detected by BeyoClick^TM^ eyoClick cell proliferation detection kit (Biyuntian, C0078S). The following are the general steps. Add EdU to the cell culture medium at a final concentration of 10 μM, incubate for 10 h, and fix with 4% paraformaldehyde for 15 min after phosphate-buffered saline (PBS) immersion. Remove the fixative solution by PBS immersion, and then incubate with 0.5% Triton X-100 at room temperature for 15 min to permeate the membrane. After removing the permeabilization solution, immerse in PBS, add 0.5 ml of Click reaction solution, and incubate for 30 min at room temperature in the dark. Remove the reaction solution, after immersion in PBS, add Hoechst 33342 reaction solution dropwise and incubate at room temperature for 15 min in the dark. Mount the slide with mounting solution containing anti-fluorescence quencher, and then observe and collect the images under a fluorescence microscope (Olympus, BX53).

### Flow Cytometry Analysis

After cell transfection, SKOV3 and OVCAR-3 cells were harvested for flow cytometry analysis. Differential groups of cells were washed with PBS three times and then stained with propidium iodide (PI)/RNase solution following the protocol of the manufacturer. Cell cycles were detected by using FACScan flow cytometer (BD Biosciences) and analyzed using FlowJo software.

### Transwell Assay

We evaluated the effect of NTPCR on the cell invasion and migration abilities by using the Transwell system. The membranes of inserts were precoated with Matrigel for invasion assay overnight. After that, SKOV3 and OVCAR-3 cells were harvested and suspended using serum-free RPMI-1640 medium. Thus, 200 μl of cell suspension including 2.0 × 10^4^ cells was added into the upper chamber, while 500 μl of culture medium with 10% fetal bovine serum (FBS) was added into the lower chamber. Following incubation for 24 h, the cells were fixed with 4% paraformaldehyde and stained with 0.1% crystal violet for 10 min. The results were analyzed by using a microscope (Olympus IX73; Olympus Corporation, Tokyo, Japan).

### Western Blotting

The protein samples prepared in ice-cold RIPA buffer were separated on SDS-PAGE gels and then transferred onto polyvinylidene fluoride (PVDF) membranes. The membranes were then blocked in 5% skimmed milk at room temperature for 1 h and then incubated with the primary antibody solution (NTPCR: Biodragon BD-PT3202 anti-rabbit monoclonal, 1:1,000; GAPDH: Proteintech 60004-1-lg mouse monoclonal, 1:1,000) at 4°C overnight. The following day, the secondary antibodies (Peroxidase AffiniPure Goat Anti-Rabbit IgG (H + L), 1:10,000; Peroxidase AffiniPure Goat Anti-Mouse IgG (H + L), 1:5,000) were added and then incubated at room temperature for 2 h. For chemiluminescence development, Millipore ECL system was used; to perform grayscale analysis on the data, TanonImage was used, and then statistical analysis was performed on the grayscale analysis results. The data mapping software is GraphPad Prism5.

### Transcriptomic Sequencing

We performed RNA-sequencing on an Illumina system for shNTPCR and control shNC group with three duplications per group. Firstly, Trimmomatic version 3.6 (Bolger et al., 2014) tool was used to clean up the reads by removing adapters and low-quality reads (quality value less than 20), and the reads with N ratio exceeding 10% or length less than 50 bp were also removed. Hisat2 software (v2.05) (Siren et al., 2014) was used for the alignment of clean reads to human genomic database (GRCH38, Gencode) (Harrow et al., 2012). Moreover, human genome annotation in the resulting files was mapped to corresponding read count by using featureCounts tools (Liao et al., 2014). Further filtering was performed for different results according to the fragments per kilobase of exon per million fragments mapped (FPKM) method, and the genes with FPKM value less than 0.1 in any samples were removed.

Quasi-likelihood *F*-tests in edgeR software (Lun et al., 2016) were used to analyze DEGs between shNTPCR and shNC samples by setting | logFC| > 1.585 and false discovery rate (FDR) < 0.05 as cutoff criteria. Two-dimensional clustering analysis and functional enrichment analysis were conducted for these DEGs.

We further screened the gene–disease associations from the DisGeNET database (Piñero et al., 2016), a comprehensive platform containing information on human disease genes and related variants. As for the DEGs screened from the database, the expression status in EOC was investigated by using Gene Set Enrichment Analysis (GSEA) algorithm, a computational algorithm to assess significant differences of defined gene sets among biological phenotypes (Subramanian et al., 2007).

We analyzed the interactions of protein–protein according to the STRING online tool and constructed the protein–protein interaction (PPI) network using Cytoscape software. It has been shown that proteins with similar functions tend to cluster together in the PPI network, and thus, we mined the functional modules using MCODE tool (Bader and Hogue, 2003; Davis, 2015).

To further explored the regulation mechanism of EOC, we screened the miRNA–gene and TF–gene pairs using Enrichr (Chen et al., 2013) and ENCODE tools (Rosenbloom et al., 2011) and constructed an miRNA–TF–gene network.

### Metabolomics Sequencing

The EOC cell extract samples derived from the shNTPCR and shNC groups were analyzed by the ultra performance liquid chromatography (UPLC) method on a quadrupole time-of-flight (Q-TOF) liquid chromatography–mass spectrometry (LC-MS) system. Duplicates from 10 controls together with five QC samples were obtained to evaluate the assay reproducibility.

Metabolomics data under positive ion mode and negative ion mode were obtained and first normalized before multivariate analysis. We performed unsupervised principal component analysis (PCA) and supervised partial least-square discriminant analysis (PLS-DA) to explore the metabolic differences in NTPCR samples.

As for the differential performance of metabolites between two groups, metabolite analysis was conducted using MBROLE 2.0, which is a web server designed to permit systemic analysis for metabolomic data (Chong et al., 2018). Corresponding KEGG pathways were screened with *p* < 0.05 as cutoff criteria.

### Integrated Transcriptomic Analysis and Metabolomics Analysis

We used the KEGG pathway-based method to analyze differential metabolites and genes in a biological process. The thresholds were set as num-overlapping-metabolites/genes > 0 and p joint/metabolites < 0.05.

### Statistical Analysis

Prism9.0 statistical software was used for data analysis. All data were expressed as mean ± standard deviation (±S). Pairwise comparisons between different groups were performed by one-way analysis of variance, and *p* < 0.05 was considered statistically significant.

## Results

### NTPCR Was Upregulated in EOC Tissues

**FIGURE 1:**
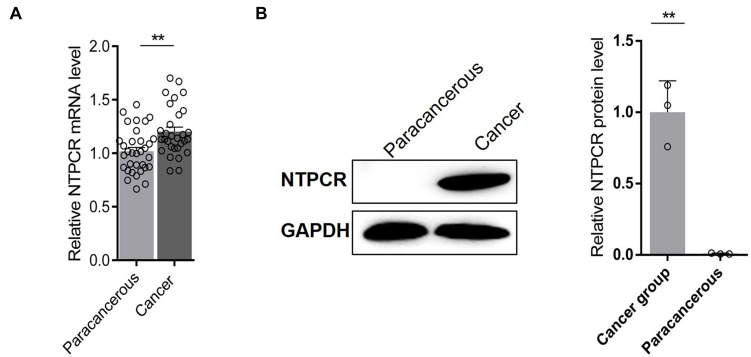
*Via***(A)** RT-qPCR analysis and **(B)** Western blot analysis, the expression of NTPCR in cancer tissues was significantly increased compared to para-cancerous tissues (**p < 0.01).

The expressed status of NTPCR was detected in EOC tissues via RT-qPCR analysis and Western blot analysis. The results showed that the expression of NTPCR in cancer tissues was significantly increased compared to para-cancerous tissues (Figure 1, *p* < 0.05).

### NTPCR Inhibits Cell Proliferation, Cell Cycle, and Cell Migration and Invasion in the EOC Cell Line

**FIGURE 2:**
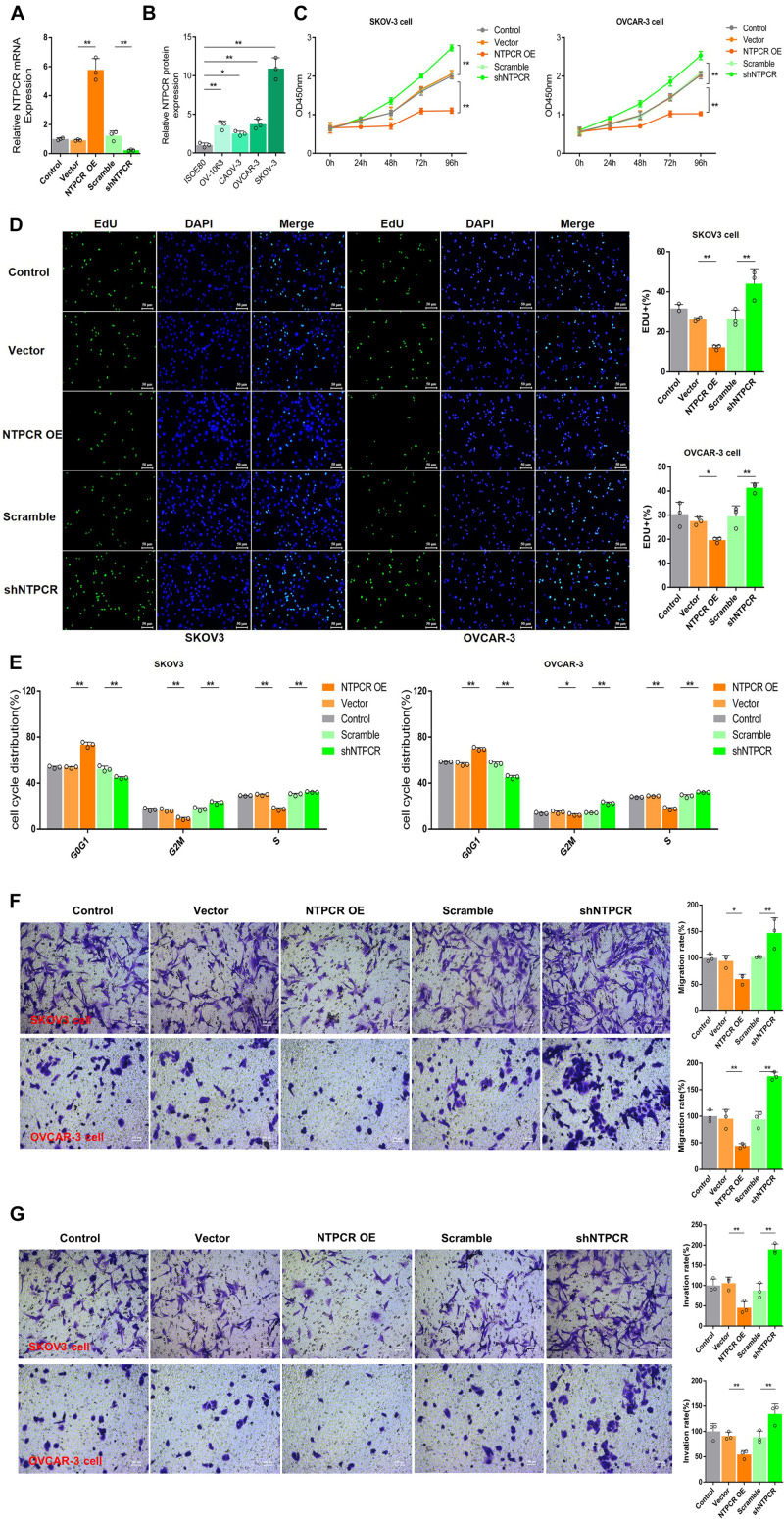
Knocking out of NTPCR-induced cell proliferation, S phase arrest, promoted cell invasion, and migration in SKOV3 cell line. **(A)** The construction of the overexpression vector and interference shRNA vector of NTPCR. It can be seen from the left figure that compared with Scramble groups, the expression of NTPCR in shRNA groups was significantly reduced, and shRNA1 was more obvious. That is why we previously chose shRNA1 for the subsequent experimental studies. It also can be seen from the right figure that compared with the vector groups, the expression of NTPCR in NTPCR OE groups was significantly increased, and compared to the Scramble groups, shNTPCR groups were significantly reduced (*p < 0.05, **p < 0.01). **(B)** NTPCR expression levels were significantly upregulated in four ovarian cancer cell lines (SKOV3 vs. IOSE80, *p* < 0.01; CAOV-3 vs. IOSE80, *p* < 0.05; OV-1063 vs. IOSE80, *p* < 0.01; and OVCAR-3 vs. IOSE80, *p* < 0.01) compared with the human ovarian epithelium cell line IOSE80. **(C)** The effect of NTPCR on the viability of epithelial ovarian cancer cells. The CCK-8 assays were performed to evaluate the cell proliferation after being cultured at 24, 48, 72, and 96 h (**p < 0.01). **(D)** The effect of NTPCR on the DNA replication ability of epithelial ovarian cancer cell lines SKOV3 and OVCAR-3. In the figure, EdU detects the DNA replication ability (*p < 0.05, **p < 0.01). **(E)** The picture shows the distribution of the cell cycle detected by PI. NTPCR increased the proportion of G0/G1-phase cells in epithelial ovarian cancer cells and decreased the proportion of S-phase and G2/M-phase cells (*p < 0.05, **p < 0.01). **(F,G)** Transwell assesses the effect of NTPCR on the migration and invasion of epithelial ovarian cancer cells. In SKOV3 cells and OVCAR-3 cells, overexpression of NTPCR reduced cell migration ability, and downregulation of NTPCR increased cell migration ability. The results show the same in the cell invasion experiment (*p < 0.05, **p < 0.01).

To further investigate the potential role of NTPCR on the biological function of EOC, we first constructed the overexpression and interference vector of NTPCR, and qRT-PCR verified its effect (Figure 2A). Simultaneously, analysis of NTPCR expression levels in different EOC cell lines (SKOV3, CAOV-, OV-1063, and OVCAR-3), and normal ovarian epithelial cell line IOSE80 as a control, found that the expression levels of NTPCR in SKOV3 and OVCAR-3 cells were higher (Figure 2B, *p* < 0.05); hence, we chose these two strains of cells for follow-up experiments.

In the CCK-8 assays, cell viability exhibited significant differences between NTPCR over expression (OE) and shNTPCR groups (Figure 2C, *p* < 0.01), indicating that overexpression of NTPCR inhibited EOC cell viability and interference with NTPCR expression increased cell viability. EdU results showed that NTPCR downregulation of NTPCR expression promotes the DNA replication process (Figure 2D), which shows that knockdown of NTPCR promoted cell proliferation in EOC cells. The effect of NTPCR on the cell cycle was also analyzed by flow cytometry analysis, and we found that overexpression of NTPCR increased the proportions of G0/G1phase cells and decreased the proportions of S- and G2/M-phase cells, while downregulating NTPCR expression was just the opposite (Figure 2E). Finally, through the Transwell chamber experiment, we found that NTPCR inhibited the metastatic and invasive abilities of EOC cells (Figures 2F,G). On the whole, we have enough reasons to believe that NTPCR can inhibit the biological process of EOC and act as a tumor suppressor.

### Transcriptomic Analysis on the Effect of NTPCR Expression in EOC

**FIGURE 3:**
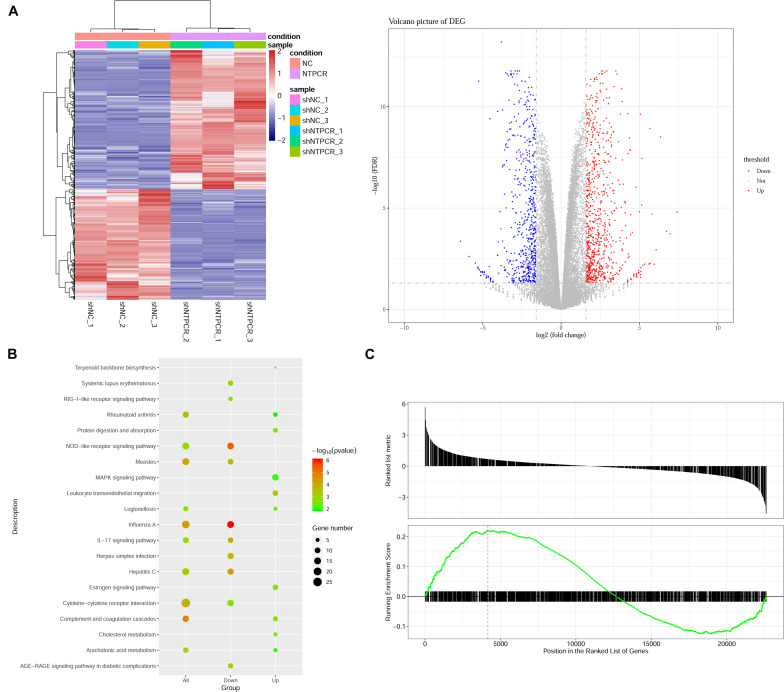
Identification of DEGs between shNTPCR ovarian cancer groups and control shNC groups. **(A)** Two-dimensional clustering analysis (left) and volcano plot (right) for DEGs. Columns in the clustering analysis results represent different samples, while rows refer to differential genes; red and blue color represent upregulated and downregulated expression. Dots in the volcano plot diagram represent differential genes. Red and blue dots refer to upregulated and downregulated genes. **(B)** KEGG pathway enrichment analysis results for DEGs. The pathway with a smaller *p*-value represented a significant enrichment degree. The dot sizes refer to the count number of genes enriched by the pathway category. **(C)** GSEA results of dysregulated genes in ovarian cancer.

On the basis of clarifying that NTPCR inhibits the biological process of EOC, we intended to initially explore the mechanism of NTPCR regulating the progress of EOC. First, we analyzed the mRNA expression profile of the shNTPCR and shNC groups through transcriptomics and screen out DEGs, and under the cutoff criteria of | log_2_FC| > 1.585 and FDR < 0.05, we screened a total of 1,408 DEGs between the shNTPCR group and shNC group, including 783 upregulated and 625 downregulated genes. Bidirectional clustering analysis and Volcano Plot for DEGs are shown in Figure 3A.

We performed the Gene Oncology (GO) and Kyoto Encyclopedia of Genes and Genomes (KEGG) pathway enrichment analysis for DEGs in shNTPCR groups. The results showed that these genes were mainly related to biological processes of response to the virus, viral genome replication, response to lipopolysaccharide, and extracellular matrix organization. The signaling pathway categories included NOD-like receptor signaling pathway, IL-17 signaling pathway, hepatitis C, and cytokine–cytokine receptor interaction (Figure 3B).

Moreover, we analyzed the dysregulated gene sets in the shNTPCR group samples by using the GSEA algorithm. The results are shown in Figure 3C (enrichment score = 0.221325, *p* < 0.05). The enrichment score for upregulated genes is slightly higher than that of downregulated genes, indicating that the expression status of gene sets might exhibit a similar effect on EOC.

### PPI Network Analysis

**FIGURE 4:**
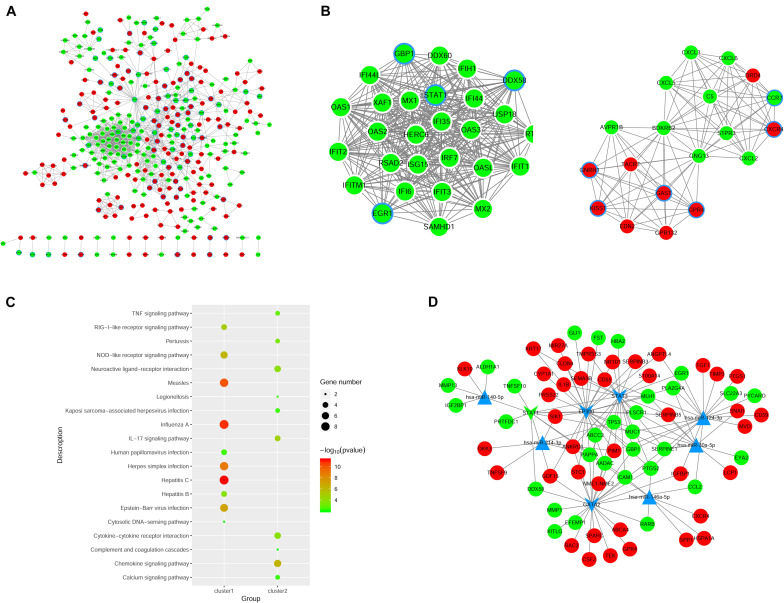
Regulatory network analysis to predict miRNAs and TFs associated with ovarian cancer. **(A)** PPI network construction to analyze DEGs in shNTPCR ovarian cancer groups. The red node represent upregulated genes, while the green dot refer to downregulated genes. The node with blue aperture indicates genes related to ovarian carcinoma. **(B)** Two clusters of functional modules were identified from the PPI network. **(C)** Functional enrichment analysis results for DEGs in subnetwork modules, and the top 10 pathway categories were sorted according to the *p*-value. **(D)** Regulatory network of miRNA–TF–gene was constructed to predict crucial genes correlated with ovarian cancer progression. Dots in red and green color represent upregulated and downregulated genes, respectively; triangular nodes and V-shaped nodes represent miRNA and TFs.

We constructed the PPI networks for these DEGs and screened the top 10 node genes with the higher scores based on the three topological properties (Figure 4A and Table 1). There were 348 nodes and 1,154 edges in the network. Several genes obtained higher degree values, such as STAT1 (degree = 43), OAS2 (degree = 36), OAS1 (degree = 36), IFIT3 (degree = 36), OASL (degree = 36), and MX1 (degree = 36).

**TABLE 1:** The top 10 hub genes in PPI network were identified as candidate genes related to ovarian cancer according to topological properties (degree centrality, betweenness centrality, and closeness centrality).

Gene	Degree	Gene	Betweenness	Gene	Closeness
STAT1	43	TP53	30206.2	TP53	0.015058
OAS2	36	STAT1	14016.98	STAT1	0.015041
OAS1	36	SPP1	9019.094	ICAM1	0.01504
IFIT3	36	IL1B	8083.106	CCL2	0.015017
OASL	36	ICAM1	7465.445	PTGS2	0.015003
MX1	36	ITGB2	6537.538	SPP1	0.015002
OAS3	35	CMPK2	6499.484	IL1B	0.014997
TP53	34	NME1-NME2	5982	CXCR4	0.014997
IRF7	34	CYP1A1	5829.825	TLR3	0.014992
RSAD2	33	TIMP2	5323.395	EGR1	0.014985

Furthermore, we used MCODE plug to screen subnetwork modules related to PPI networks. By setting the score ≥ 10 and node ≥ 10 as cutoff criteria, two subnetwork modules were screened (Figure 4B). Several signaling pathways were identified as associated with these DEGs, including human papillomavirus infection, TNF signaling pathway, and Kaposi sarcoma-associated herpesvirus infection (Figure 4C).

### Prediction of miRNA and TFs Associated With EOC

The miRNAs and TFs correlated with DEGs were predicated by using Enrichr tools (Table 2). The top five miRNAs and TFs with the higher scores (combine score) were selected to establish a regulatory network of an miRNA–TF–target gene (Figure 4D). *Hsa-miR-124-3p* and EP300 obtained the highest count compared with other miRNAs or genes (*Hsa-miR-124-3p*, gene number = 195, *p* < 0.01; EP300, gene number = 27, *p* < 0.01), indicating the two genes were hub genes in the regulatory network.

**TABLE 2:** The top five miRNAs and transcription factors with higher combined scores were predicted to be crucial factors associated with ovarian cancer.

Term	Gene num	*P*-value	Combined score	Genes
hsa-miR-124-3p	195	0.000706	70.998557	IGFBP1, SLC22A3, EGR1, SERPINE1, PLA2G4A, PTGS2, FGF1, PTGS1, PYCARD, MUC1, SNAI1, PIM1, TIMP3, CCL2, MVD, CD59, ANGPTL4, GBP1, CD55
hsa-miR-146a-5p	7	0.000182	20.229601	STAT1, SPP1, CXCR4, RARB, PTGS2, ICAM1, HSPA1A
hsa-miR-30a-5p	9	0.028172	19.442688	PLSCR1, EYA2, SERPINE1, SNAI1, PLA2G4A, LCP1, MLH1, TP53, SERPINB5
hsa-miR-140-5p	5	0.000304	17.247252	MMP13, STAT1, ALDH1A1, IGF2BP1, KLK10
hsa-miR-214-3p	6	0.001346	17.236654	PAPPA, PIM1, TNFSF9, SIK1, TP53, DKK3
EP300	27	2.87*E*−06	22.275263	TMPRSS3, SERPINE1, SEMA3B, STC1, PTGS2, GLI1, PIM1, ANKRD1, GBP1, CD55, SERPINB3, ABCC2, GDF15, FST, PLA2G4A, NR1D1, MLH1, SERPINB5, CLDN4, AADAC, KRT17, IL1B, PAPPA, CYP1A1, SIK1, ANGPTL4, TP53
GATA2	25	0.000196	14.549745	CSF2, SPARC, SERPINE1, STC1, PTGS2, ICAM1, NME1-NME2, EFEMP1, RAC2, PIM1, ANKRD1, CCL2, GBP1, IGFBP1, ABCC2, GDF15, DDX58, MMP1, ABCA4, GPR4, KITLG, AADAC, PAPPA, RARB, TEK
EP300	6	0.000578	14.452685	STAT1, SEMA3B, ANKRD1, MIR27A, PRSS22, CD55
STAT3	24	0.000407	13.263751	SERPINB3, EGR1, ABCC2, STAT1, TMPRSS3, SERPINE1, SEMA3B, HBA2, NR1D1, PTGS2, MLH1, SERPINB5, ICAM1, NME1-NME2, MUC1, PLSCR1, KRT17, IL1B, PAPPA, PIM1, ANKRD1, S100A14, GBP1, CD55
STAT1	8	0.000966	12.789136	MUC1, PLSCR1, GDF15, STAT1, DDX58, TNFSF10, PRTFDC1, ICAM1

### Differential Metabolites Between NTPCR and Normal Samples

**FIGURE 5:**
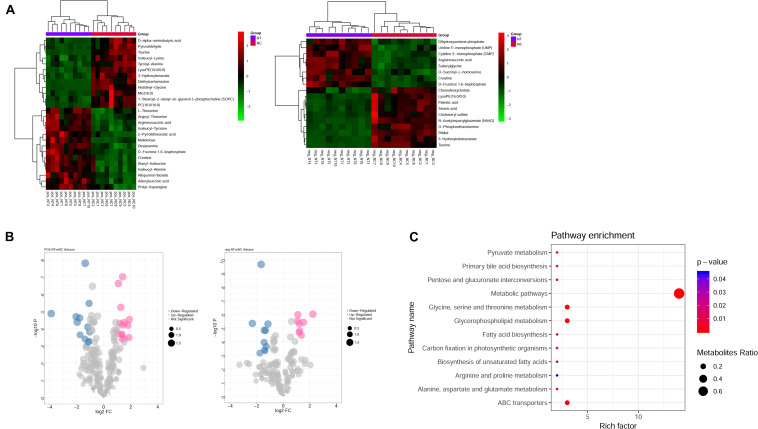
Identification of differential metabolites between NTPCR groups and normal groups. **(A)** Hierarchical clustering analysis was performed for differential metabolites in NTPCR vs. normal control samples under ESI + ion (left) and ESI- ion (right) modes. Green represent downregulated metabolites, while red refer to upregulated metabolites. **(B)** Volcano plot of differential metabolites in NTPCR vs. normal control samples under ESI + ion (left) and ESI- ion (right) modes. Blue dots are downregulated metabolites, pink dots refer to upregulated metabolites, and gray dots represent normal metabolites. The dot size was correlated with VIP values. **(C)** Functional enrichment results of differential metabolites in NTPCR vs. control groups. The horizontal axis is the count number of pathways, while the vertical axis is the pathway names. The dot size represents the ratio of pathways enriched by metabolites. The color change from blue to red means a smaller *p*-value.

Metabolism is the downstream of gene regulatory network and protein action network and is closely related to the phenotype of organisms. DEGs may ultimately affect the composition and level of intracellular metabolic spectrum. We used mass spectrometry to detect metabolite difference between the shNTPCR and shNC groups; by the threshold of FDR < 0.01, | log2FC|, and VIP > 1, we screened a total of 44 differential metabolites, including 26 metabolites in ESI + ion mode and 18 metabolites in the ESI− ion mode. Among these metabolites, there were 22 upregulated and 22 downregulated metabolites. Hierarchical cluster analysis and volcano plot results showed that these dysregulated metabolites can be significantly classified into two differential groups (Figures 5A,B).

Functional analysis results showed that a total of 12 signaling pathways were screened, including metabolic pathways, glycine, serine and threonine metabolism, glycerophospholipid metabolism, and ABC transporters (Figure 5C).

### Integrated Transcriptomic and Metabolomic Analysis

**FIGURE 6:**
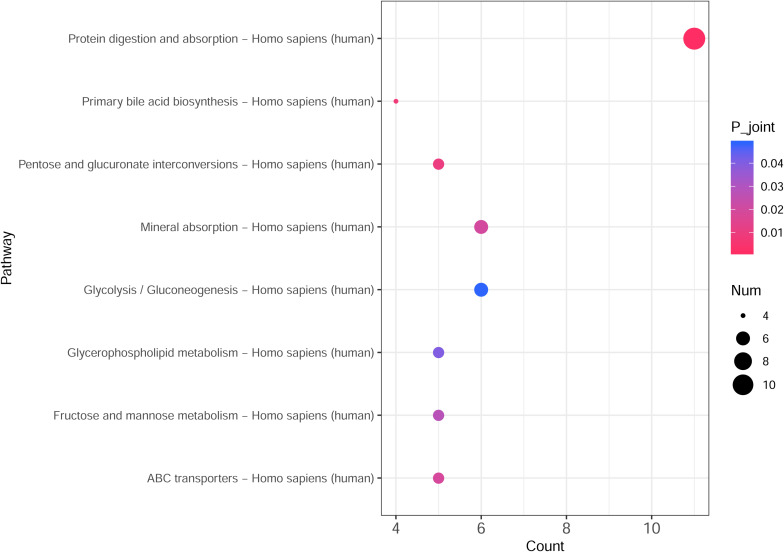
Integrated transcriptomic and metabolomic analysis results to identify crucial KEGG pathways related to ovarian cancer. The horizontal axis is the pathway categories, and the vertical axis is the count number of differential genes and metabolites. A *p* < 0.05 represents a significant difference, and the dots with the color changing from pink to red represents a smaller *p*-value.

The integrated transcriptomic and metabolomic analysis showed that DEGs and differential metabolites in the NTPCR groups were associated with eight overlapped pathways, such as primary bile acid biosynthesis, protein digestion and absorption, pentose, and glucuronate interconversions (Figure 6). Of these pathways, pentose and glucuronate interconversions obtained a higher overlapped gene number than other pathways, including 10 overlapped genes (SLC7A7, SLC7A8, COL4A5, etc.) and one metabolite (L-threonine).

## Discussion

In this study, we demonstrated the potential roles of NTPCR in EOC. It was observed that NTPCR was downregulated in EOC tissues and NTPCR can inhibit cell proliferation, migration, and invasion, and decrease the proportions of S- and G2/M-phase cells, indicating that NTPCR might serve as a tumor suppressor in ovarian cancer.

NTPCR (THEP 1, C1orf57, or HCR-NTPase) is a non-specific nucleoside triphosphatase exhibiting a slow hydrolyzed activity *in vitro*. It is overexpressed in several tumor tissues including neuroblastoma and liver cholangiocarcinoma (Placzek et al., 2007; Pasdziernik et al., 2009). A previous study reported there was no effect observed in NTPCR-silencing neuroblastoma cells, while overexpression of NTPCR led to the cytotoxicity of this protein (Pasdziernik et al., 2009). However, the expressed status and physiological role of NTPCR is still unknown in other cancer types, especially in ovarian cancer. Our study firstly reported that NTPCR might serve as a tumor suppressor in ovarian cancer progression.

RNA sequencing results revealed that disturbance of transcriptome was observed in shNTPCR ovarian cancer cells, and several miRNAs and TFs were predicted to be hub genes correlated with ovarian cancer, including hsa-miR-124-3p, hsa-miR-146a-5p, hsa-miR-30a-5p, EP300, GATA2, and STAT3. MicroRNAs are endogenous small RNAs that function as pivotal roles in tumorigenesis, and they execute the posttranscriptional regulation process by interacting with target genes (Iorio et al., 2007; Kasinski and Slack, 2011). miR-124 s highly conserved in humans. Abnormally expressed has-miR-124 has been reported in multiple cancers, such as gastrointestinal carcinomas (Xia et al., 2012; Zhang et al., 2014) and hepatocellular carcinoma (Chen et al., 2018). Down-regulation of miR-124 was identified in highly metastatic ovarian cancer cells and human tissue specimens (Zhang H. et al., 2013). Consistent with the role in gastric cancer, it could inhibit invasive and migratory abilities of ovarian cancer cells according to interaction with the 3′-untranslated (3′-UTR) regions of SphK1 mRNA. Similarly, another paper showed miR-124 might serve as a tumor suppressor in malignant tumors, and thus, upregulated miR-124 could inhibit cell proliferation and migration by decreasing PDCD6 expression in SKOV3 and OCVAR3 cells (Yuan et al., 2017). A recent paper revealed miRNA-124-3p targeting gene Gata2 might be a novel biomarker related to ovarian cancer progression based on multiomics analysis of the tumor microenvironment (Gov et al., 2017). GATA-binding factor 2 (GATA2) is a TF that is critical for many physical processes, such as embryonic development, blood forming, and tissue-forming stem cells. Abnormal GATA2 epigenetic dysregulation can induce unfavorable phenotypes in human gastric cancer by decreasing expression of GATA6, which has a vital role in gastrointestinal development (Song et al., 2018). Considering this, our study is consistent with a previous study that miR-124 might interact with target gene GATA2 to regulate the progression of ovarian cancer.

As for miR-146a, Wilczyński et al. (2017) analyzed the expression of miRNAs in ovarian cancer patients undergoing surgery and found miR-146a was increased in primary tumors compared with normal ovarian tissue (*p* = 0.02); lower levels of miR-146a in patients were correlated with shorter survival times. Many ovarian cancer patients with a strong family history had mutations of BRCA1/BRCA2, and a previous study demonstrated miR-146a can bind to the 3′-UTR region of the two genes and regulated their expression (Pastrello et al., 2010); patients who had polymorphism of hsa-mir-146a (rs2910164) may be diagnosed at a younger age than patients without a variant allele. Histone acetyltransferase p300 (EP300) is also known as p300, and it functions as a histone acetyltransferase that regulates cell growth and division. Somatic missense alterations of EP300 have been identified in gastrointestinal cancer, such as colorectal and gastric cancer (Muraoka et al., 1996). Six truncating mutations were identified in various human cancer types, including ovarian carcinoma (Gayther et al., 2000). Moreover, EP300 can be targeted by the miR-106b∼25 cluster and be involved in regulating multidrug resistance in an ABC transporter-independent manner (Hu et al., 2016). Prevalent evidence uncovered the roles of STAT3 in ovarian cancer. Elevated STAT3 expression was identified in ovarian cancer ascites and could promote invasion and metastasis (Saini et al., 2017). STAT3 also interacting with miRNA-92 promoted malignant progression in ovarian cancer, and the potential mechanism was associated with regulation of the Wnt signaling pathway (Chen et al., 2017). Multiomics profiling reveals STAT3 regulated several biological processes in ovarian cancer, including epithelial–mesenchymal transition, cell cycle progression, and E2F signaling (Lu et al., 2019).

In addition, metabolites were final products in biological processes and can be disturbed by genetic factors. The integrated transcriptomic and metabolomic analysis demonstrated that significant alterations of eight pathways were identified in the NTPCR group, such as pentose and glucuronate interconversions, primary bile acid biosynthesis, protein digestion, and absorption. A total of 10 overlapped genes (SLC7A7, SLC7A8, COL4A5, etc.) and one metabolite (L-threonine) were significantly correlated with the pathway of protein digestion and absorption in ovarian cancer. AKT serine/threonine kinase 1 can be target by microRNA-215 to regulate the progression of breast cancer (Yao et al., 2017). Moreover, serine–threonine kinases (STK) were also reported as attractive therapeutic targets in EOC; the combination of STK inhibitors with cytotoxic agents or other class biological agents significantly improved clinical benefit rates (Ciccone et al., 2016).

In summary, our study provided an experimental foundation that decreased NTPCR can affect cell proliferation, cell cycle, cell migration, and invasion in SKOV3 cells. Further experimental foundations should be conducted to investigate the mechanism of NTPCR in ovarian cancer progression. Integrative analysis of transcriptomic and metabolic profiling identified crucial pathways related to differential metabolites and genes. This study might provide attractive targets for the potential treatment of ovarian cancer patients.

## Data Availability Statement

The data can be found in online repositories: Gene Expression Omnibus GSE178486.

## Ethics Statement

The studies involving human participants were reviewed and approved by Ethics Committee of Hangzhou First People’s Hospital. The patients/participants provided their written informed consent to participate in this study.

## Author Contributions

JT: conception and design of the research. HS and ZR: acquisition of data. HZZ and ZR: analysis and interpretation of data. HS, HZZ, and HJZ: drafting the manuscript. ZZ and JT: revision of manuscript for important intellectual content. HS: conducting experiments. All authors read and approved the final manuscript.

## Conflict of Interest

The authors declare that the research was conducted in the absence of any commercial or financial relationships that could be construed as a potential conflict of interest.

## Publisher’s Note

All claims expressed in this article are solely those of the authors and do not necessarily represent those of their affiliated organizations, or those of the publisher, the editors and the reviewers. Any product that may be evaluated in this article, or claim that may be made by its manufacturer, is not guaranteed or endorsed by the publisher.

## Abbreviations

EOC: epithelial ovarian carcinoma
NTPCR: nucleoside-triphosphatase cancer-related
DEGs: differential expressed genes
NGS: next-generation sequencing
TFs: transcription factors
GO: Gene Oncology
KEGG: Kyoto Encyclopedia of Genes and Genomes
GATA2: GATA-binding factor 2
EP300: histone acetyltransferase p300
STK: serine-threonine kinases
PI: propidium iodide
FPKM: fragments per kilobase of exon per million fragments mapped
GSEA: Gene Set Enrichment Analysis
UPLC: ultra performance liquid chromatography
PCA: principal component analysis
PLS-DA: partial least-square discriminant analysis.
